# The magnitude of COVID-19 related stress, anxiety and depression associated with intense mass media coverage in Saudi Arabia

**DOI:** 10.3934/publichealth.2020052

**Published:** 2020-09-01

**Authors:** Yosef Mohamed-Azzam Zakout, Fayez Saud Alreshidi, Ruba Mustafa Elsaid, Hussain Gadelkarim Ahmed

**Affiliations:** 1 Department of Pathology, College of Medicine, University of Hail, Hail, Kingdom of Saudi Arabia; 2 Department of Family and Community Medicine, College of Medicine, University of Hail, Hail, Kingdom of Saudi Arabia; 3 Department of Histopathology and Cytology, Faculty of Medical Laboratory Sciences, University of Khartoum, Khartoum, Sudan

**Keywords:** anxiety, COVID-19, depression, media, Saudi Arabia, stress

## Abstract

The Coronavirus Disease 2019 (COVID-19) has resulted in catastrophic consequences in many aspects of life; including negative psychological effects. We aimed to assess the mental health status of a group of Saudi population during this pandemic. Also, we aimed to assess the effect of the intensive media coverage of the pandemic news on mental health status. A questionnaire was distributed online to test depression, anxiety, and stress (using the DASS-21 scale) and their relationship to certain variables. A total of 215 respondents were included. Higher mental health prevalence rates were reported in non-Saudi participants compared to Saudi ones (i.e., 50.74% vs. 30.40%; 34.23% vs. 13.51% and 59.70% vs. 27.70%; for depression, anxiety and stress, respectively). About 55.8% of the participants felt the extensive coverage in the media of COVID-19 news, which caused higher mental sufferings. Higher mental health prevalence rates were reported in females compared to males participants (i.e., 56.97% vs. 23.25%; 30.23% vs. 13.17% and 54.65% vs. 26.35%; for depression, anxiety and stress, respectively). Reasonable following of the COVID-19 news; and less exposure to the pandemic information could help in reducing the mental health issues related to the ongoing pandemic. Special care and attention should be paid to females and younger people who seem to be particularly affected during the era of COVID-19.

## 1. Introduction

Firstly, the COVID-19 was originated in Wuhan, China in late 2019, which subsequently spread throughout the entire world after a very short period of time [1]. It is caused by the severe acute respiratory syndrome coronavirus 2 (*SARS*-*CoV*-*2*), which is the causative agent of this disease that represents a worldwide public health issue [2]. Regarding the symptoms in patients with pneumonia caused by SARS-CoV-2 (novel coronavirus pneumonia); fever is the most common one, followed by cough, dyspnea, headache, and diarrhea, respectively [3].

This virus has affected the physical health of millions of people globally and was expected to cause mental health issues [4]. It causes an increase in negative emotions including indignation, depression, and anxiety [5]. For this reason, China applied emergency psychological crisis interventions to minimize the negative psychological effects on public mental health because of COVID-19 [6]. Therefore, it is essential to integrate public mental health interventions into emergency response and public health intervention plans [6]. The population estimates in the Kingdom of Saudi Arabia in 2018 show that Saudis represent 62.15% and non-Saudis 37.84%. Males make up 57.58% and females 42.41% of the entire population [7]. Several studies have tested the psychological and mental health status during the COVID-19 pandemic [8]–[12]. For instance, Wang et al. (2020) studied public psychological states during the outbreak of COVID-19. They reported that out of 600 participants; anxiety and depression were detected in 6.33% and 17.17% in this order [13]. Cao et al. (2020) tested the psychological impact of COVID-19 on 7,134 Chinese college students. They found that 0.9% of the study subjects were experiencing severe anxiety, 21.3% mild anxiety, and 2.7% moderate anxiety [14]. Liu et al. (2020) reported that attention should be paid to public psychological stress during the COVID-19 epidemic; particularly in young individuals who seemed likely to experience psychological issues [15]. The study of mental health issues during COVID-19 pandemic is essential; because it can be associated to severe psychological impacts such as suicide [16]–[18]. Additionally, the misinformation regarding the current pandemic, mainly in social media, might affect people's mental health including anxiety and depression [18]. Accordingly, it was suggested to avoid unreliable sources of information and news such as social media [18]. Therefore, the current study aims to provide a picture of the mental health status of a group of Saudis and non-Saudis, most of them were living in the Kingdom of Saudi Arabia during the pandemic, which provides valuable information for mental health care officials to develop a mental health care policy that takes into account the most affected groups during the current pandemic. The study also aims to assess the relationship between intensive follow-up to pandemic news and mental issues and their severity. It also help identify the sources of information that most participants rely on in following the pandemic news and information; which may help the health care officials in identifying the most effective platform to provide reliable information regarding this pandemic and mitigate its psychological effects.

## 2. Materials and methods

### 2.1. Participants

The study included Saudi participants of different ages, gender, educational levels, and occupations. Similarly, non-Saudis, majority are living in Saudi Arabia, were included too.

### 2.2. Survey-procedures

A questionnaire was prepared and distributed online using different tools including WhatsApp, Twitter, and electronic mails. The participants were reached by distributing the questionnaire in different groups of WhatsApp, posting it on twitter and sending electronic emails. Information regarding this study was included at the beginning of the online questionnaire and the participants were informed that by filling and sending this questionnaire they agree to take part in this study and the results will be used only for scientific and research purposes. The study included Saudi participants of different ages, gender, educational levels, and occupations. Similarly, non-Saudis, majority are currently living in Saudi Arabia, were included too.

The collection of responses was between 28 May and 1 June 2020. All respondents over this period were included in the study. 101 responses received on day one, 92 on day two, 16 on day three, 4 on day five and 2 in day six. The first response was at 3:39 pm on day 1 and the last response was at 4:57 on day six. The online questionnaire covered socio-demographic data; frequency of following the news of COVID-19; primary news source; a direct question whether or not the participants feel the extensive coverage in the media of COVID-19 news causes them stress and/or anxiety; whether the participant is living alone; in addition to another 21 questions to assess the mental health status.

### 2.3. Measurements

**i. Socio-demographic:**

The socio-demographics were regarding nationality, whether the participant is in KSA, gender, age, marital status, educational level, and occupation.

**ii. COVID-19 media coverage:**

The extent of following the pandemic news options included excessively (daily), actively (4–6 days a week), moderately (2–3 days a week), and rarely (1 day or less weekly). The primary source of the pandemic news options included TV, radio, social media, journals, and the internet.

**iii. DASS-21:**

The mental health status was assessed using the Arabic version of Depression, Anxiety, and Stress scale (DASS-21) [19]. This scale contains 21 questions. Every 7 questions test one of the three mental health status items. The subscale of depression is composed of questions 3, 5, 10, 13, 16, 17, and 21. The subscale of anxiety composed of questions 2, 4, 7, 9, 15, 19, and 20. Finally, the subscale of stress includes questions 1, 6, 8, 11, 12, 14, and 18. As for depression subscale, the total score of the 7 questions was divided into normal when the score (multiplied by two) is between 0–9, mild depression 10–12, moderate depression 13–20, severe depression 21–27, and extremely severe depression 28–42. For anxiety subscale, the total score of the 7 questions was divided into normal when the score (multiplied by two) is between 0–6, mild anxiety 7–9, moderate anxiety 10–14, severe anxiety 15–19, and extremely severe anxiety 20–42. Concerning stress subscale, the total score of the 7 questions of this item divided into normal when the score (multiplied by two) is between 0–10, mild stress 11–18, moderate stress 19–26, severe stress 27–34 and extremely severe stress 35–42 [20].

DASS-21 scale seems to be useful in evaluating the mental health status in several publications and populations, including the Saudi population [21]–[28]. Also, this scale was used in a previous study related to the COVID-19 pandemic [20].

### 2.4. Ethics

Ethical approval was obtained from the ethical committee, College of Medicine, University of Hail. Approval number: HREC 00124/CM-UOH.04/20.

### 2.5. Statistical procedure

Collected data were analyzed using IBM SPSS statistics and MedCalc® [29]. Descriptive statistics were performed for socio-demographics. Also, the means of DASS-21 subscales and the standard deviations were calculated. Odd ratio (OR) and chi-square tests were calculated with a significant level of P value less than 0.05 for chi-square tests.

## 3. Results

The responses of 215 subjects were included in this study. The mean age was 35.75 years and most responses were from the Kingdom of Saudi Arabia (KSA). In which, 205 (95.3%) were currently living in KSA compared to 10 (4.7%) outside KSA (7 Saudi and 3 non-Saudi). 148 (68.8%) of the study population were Saudis and 67 (31.2%) were non-Saudis. The male-female ratio was 1.5:1 as shown in Table 1, which shows the socio-demographics of the study subjects.

The means of depression, anxiety, and stress of the current study population were within the normal range at the DASS-21 scale (Means: 8.39, 4.09, and 9.91. Std. errors: 0.629, 0.416 and 0.656 consecutively) as shown in Table 2.

Most of the study subjects (69.3%) were found to be using social media as their primary source of the COVID-19 pandemic news. More than half of the study population, 120 (55.8%), answered (Yes) when they were asked if they feel the extensive coverage in the media of COVID-19 news causes them stress and/or anxiety.

**Table 1.** Socio-demographic characteristics of the study population.

Variable	Gender	
	Male	Female
Nationality		
Saudi	112	36
Non-Saudi	17	50
Total	129	86
Age groups (years)		
30>	24	17
31–35	44	40
36–40	26	18
41+	35	11
Total	129	86
Marital status		
Married	107	61
Unmarried	22	25
Total	129	86
Education level		
Basic study	10	6
Graduate	42	56
Postgraduate	77	24
Total	129	86
Occupation		
Unemployed	7	2
Housewife	0	12
Student	9	9
Employee	95	56
Self-employed	10	0
Retired	2	0
Other	6	7
Total	129	86

**Table 2.** The means of depression, anxiety, and stress in the study population.

Case type	Mean	Std. error of mean
Depression	8.39	0.629
Anxiety	4.09	0.416
Stress	9.91	0.656

**Figure 1.**
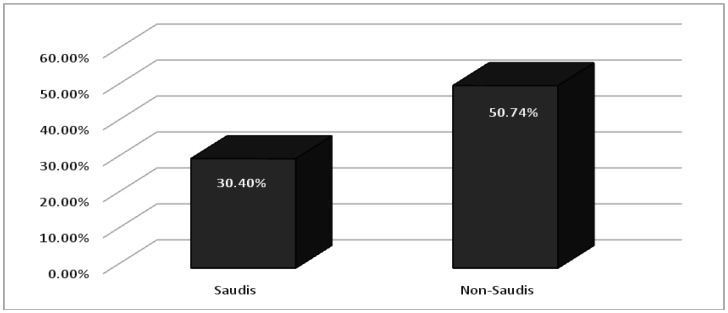
Percentages of depression among Saudi and non-Saudi participants.

As shown in Table 3; depression, of different degrees, was detected in 45 (30.40%) of Saudi participants and 34 (50.74%) in non-Saudi individuals (Figure 1). Depression was significantly higher among females 49 (56.97%) compared to males 30 (23.25%), *p-value 0.000*. Interestingly, depression was significantly higher among those who agreed that the extensive media coverage of COVID-19 news causes them stress and/or anxiety. Of whom, 56 (46.66%) were found with different degrees of depression compared to only 23 (24.21%) in those who answered (No) to this question (*p-value 0.001*).

**Table 3.** The association between variables and depression.

Variable	Depression		OR	95% CI	p-value (1-sided)
	Present (n/row %)	Absent			
Sex					
Females	49 (56.97%)	37			0.000
Males*	30 (23.25%)	99	4.370	2.4205–7.8905	
Total	79	136			
Age (years)					
Up to 35	52 (41.6%)	73	1.6621	0.9360–2.9515	
36+*	27 (30%)	63			
Total	79	136			
Nationality					
Saudi	45 (30.40%)	103			
Non-Saudi	34 (50.74%)	33			
Total	79	136			
Marital status					
Unmarried	22 (46.80%)	25			
Married*	57 (33.92%)	111	1.7137	0.8893–3.3023	0.075
Total	79	136			
Educational level					
Basic study	4 (25%)	12			
Graduate	46 (46.93%)	52			
Postgraduate	29 (28.71%)	72			
Total	79	136			
Occupation					
Unemployed	2 (22.22%)	7			
Housewife	6 (50%)	6			
Student	7 (38.88%)	11			
Employee	55 (36.42%)	96			
Self-employed	4 (40%)	6			
Retired	0 (0%)	2			
Other	5 (38.46%)	8			
Total	79	136			
Primary source of following the news of COVID-19					
Social media/Internet	72 (38.70%)	114			
T.V/journals*	7 (24.13%)	22	1.9850	0.8068–4.8835	
Total	79	136			
Do you feel the extensive coverage in the media of COVID-19 news causes you stress and/or anxiety?					
Yes	56 (46.66%)	64			
No*	23 (24.21%)	72	2.7391	1.5172–4.9452	0.001
Total	79	136			
Living alone					
Yes	4 (20%)	16			
No*	75 (38.46%)	120	0.4000	0.1288–1.2420	0.079
Total	79	136			
Daily/non-daily COVID-19 news following					
Daily followers	36 (34.61%)	68			
Non-daily followers*	43 (38.73%)	68	0.8372	0.4802–1.4597	0.314
Total	79	136			

Note: * Reference category for OR.

**Figure 2.**
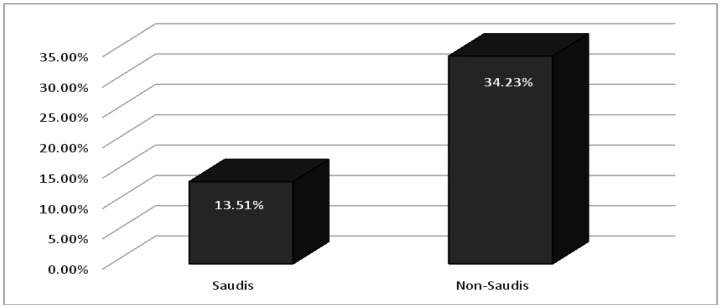
Percentages of anxiety among Saudi and non-Saudi participants.

As demonstrated in Table 4; anxiety, of different levels, was detected in 20 (13.51%) of Saudi individuals and 23 (34.23%) in non-Saudi participants (Figure 2). Also, anxiety was significantly higher among females 26 (30.23%) compared to males 17 (13.17%), *p-value 0.002*. Anxiety was significantly higher among those who agreed that the extensive media coverage of COVID-19 news causes them stress and/or anxiety. Of whom, 36 (30%) were found with different degrees of anxiety compared to only 7 (7.36%) among those who answered (No) to this question (*p-value 0.000*).

**Table 4.** The association between variables and anxiety.

Variable	Anxiety		OR	95% CI	p-value (1-sided)
	Present (n/row %)	Absent			
Sex					
Females	26 (30.23%)	60			0.002
Males*	17 (13.17%)	112	2.8549	1.4362–5.6752	
Total	43	172			
Age (years)					
Up to 35	27 (21.6%)	98	1.2742	0.6403–2.5357	
36+*	16 (17.77%)	74			
Total	43	172			
Nationality					
Saudi	20 (13.51%)	128			
Non-Saudi	23 (34.32%)	44			
Total	43	172			
Marital status					
Unmarried	13 (27.65%)	34			
Married*	30 (17.85%)	138	1.7588	0.8298–3.7281	0.103
Total	43	172			
Educational level					
Basic study	4 (25%)	12			
Graduate	29 (29.59%)	69			
Postgraduate	10 (9.90%)	91			
Total	43	172			
Occupation					
Unemployed	1 (11.11%)	8			
Housewife	2 (16.66%)	10			
Student	3 (16.66%)	15			
Employee	32 (21.19%)	119			
Self-employed	2 (20%)	8			
Retired	0 (0%)	2			
Other	3 (23.07%)	10			
Total	43	172			
Primary source of following the news of COVID-19					
Social media/Internet	36(19.35%)	150			
T.V/Journals*	7(24.13%)	22	0.7543	0.2991–1.9023	
Total	43	172			
Do you feel the extensive coverage in the media of COVID-19 news causes you stress and/or anxiety?					
Yes	36 (30%)	84			
No*	7 (7.36%)	88	5.3878	2.2729–12.7713	0.000
Total	43	172			
Living alone					
Yes	4 (20%)	16			
No*	39 (20%)	156	1.0000	0.3165–3.1597	0.597
Total	43	172			
Daily/non-daily COVID-19 news following					
Daily followers	21 (20.19%)	83			
Non-daily followers*	22 (19.81%)	89	1.0235	0.5245–1.9974	0.540
Total	43	172			

Note: * Reference category for OR.

The percentage of severe + anxiety was particularly higher in the daily COVID-19 news followers compared to the non-daily followers (9.61% vs. 3.60%, respectively, OR = 2.8457; 95% confidence interval, 0.8638–9.3748) as shown in Table 6. 8 (80%) of the severe + anxiety cases in daily COVID-19 news followers were females, 8 (80%) were of age group (up to 35 years), 8 (80%) used social media as a primary source of the COVID-19 pandemic news, 8 (80%) were non-Saudis and 10 (100%) answered (Yes) when they were asked if they feel the extensive coverage in the media of COVID-19 news causes them stress and/or anxiety.

**Figure 3.**
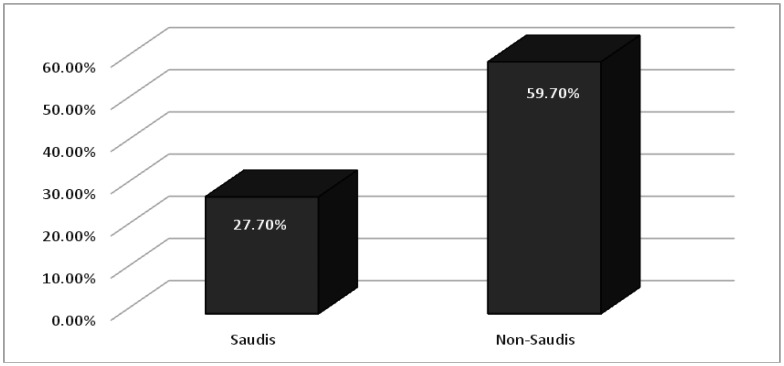
Percentages of stress among Saudi and non-Saudi participants.

As shown in Table 5, stress, of different levels, was found in 41 (27.70%) of Saudi participants and 40 (59.70%) in non-Saudi individuals (Figure 3). Stress was significantly higher among females 47 (54.65.23%) compared to males 34 (26.35%), *p-value 0.000*. Stress was significantly higher among those who agreed that the extensive media coverage of COVID-19 news causes them stress and/or anxiety; 63 (52.5%) of them were found to have different degrees of stress compared to only 18 (18.94%) among those who answered (No) to this question (*p-value 0.000*).

Stress was particularly higher in age group (up to 35 years) compared to (36+ years), (47.2% vs. 24.44%, respectively, OR = 2.7631; 95% confidence interval, 1.5235–5.0113).

**Table 5.** The association between variables and stress.

Variable	Stress		OR	95% CI	p-value (1-sided)
	Present (n/row %)	Absent			
Sex					
Females	47 (54.65%)	39	3.3673	1.8898–5.9999	0.000
Males*	34 (26.35%)	95			
Total	81	134			
Age (years)					
Up to 35	59 (47.2%)	66	2.7631	1.5235–5.0113	
36+*	22 (24.44%)	68			
Total	81	134			
Nationality					
Saudi	41 (27.70%)	107			
Non-Saudi	40 (59.70%)	27			
Total	81	134			
Marital status					
Unmarried	19 (40.42%)	28			
Married*	62 (36.90%)	106	1.1601	0.5987–2.2480	0.391
Total	81	134			
Educational level					
Basic study	4 (25%)	12			
Graduate	45 (45.91%)	53			
Postgraduate	32 (31.68%)	69			
Total	81	134			
Occupation					
Unemployed	3 (33.33%)	6			
Housewife	6 (50%)	6			
Student	5 (27.77%)	13			
Employee	57 (37.74%)	94			
Self-employed	4 (40%)	6			
Retired	0 (0%)	2			
Other	6 (46.15%)	7			
Total	81	134			
Primary source of following the news of COVID-19					
Social media/Internet	74 (39.78%)	112			
T.V/Journals*	7 (24.13%)	22	2.0765	0.8444–5.1064	
Total	81	134			
Do you feel the extensive coverage in the media of COVID-19 news causes you stress and/or anxiety?					
Yes	63 (52.5%)	57			
No*	18 (18.94%)	77	4.7281	2.5286–8.8407	0.000
Total	81	134			
Living alone					
Yes	9 (45%)	11			
No*	72 (36.92%)	123	1.3977	0.5527–3.5345	0.316
Total	81	134			
Daily/non-daily COVID-19 news following					
Daily followers	43 (41.34%)	61			
Non-daily followers*	38 (34.23%)	73	1.3542	0.7788–2.3547	0.175
Total	81	134			

Note: * Reference category for OR.

**Table 6.** The association between the daily following of COVID-19 news and severe + depression, anxiety, and stress.

Daily follower (Yes/ No)	Severe + depression		OR	95% CI	p-value (1-sided)
	Present (n/row %)	Absent			
Daily followers	12 (11.53%)	92	1.6793	0.6575–4.2895	0.196
Non-daily followers*	8 (7.20%)	103			
Total	20	195			
	Severe + Anxiety			
Daily followers	10 (9.61%)	94			
Non-daily followers*	4 (3.60%)	107	2.8457	0.8638–9.3748	0.065
Total	14	201			
	Severe + stress			
Daily followers	9 (8.65%)	95	1.4075	0.5044–3.9275	0.346
Non-daily followers*	7 (6.30%)	104			
Total	16	199			

Note: * Reference category for OR.

## 4. Discussion

During the COVID-19 pandemic, we aimed to detect the frequency and distribution of depression, anxiety, and stress in Saudi participants and non-Saudi ones. Furthermore, the study tested the association between the extensive coverage of media to the news of COVID-19 pandemic and these mental health issues. We detected that stress and depression were the most common frequent issues (37.67% and 36.74%) compared to the anxiety which was detected in 20% of the study population. These relatively high percentages of stress and depression seem to be directly related to the extensive coverage of the pandemic news in different types of media. For instance, more than half of the participants in this study (55.8%), felt that this coverage causes them stress and/or anxiety. Also, all estimated parameters were significantly higher among this group.

Interestingly, traditional media such as TV seem to be overcome by the internet-based media; 69.3% of the study subjects rely on social media as the primary source for following the COVID-19 pandemic news. Misinformation and news on social media regarding the pandemic might affect people's mental health; therefore, it was suggested to avoid unreliable sources of information and news such as social media [18]. However, this can be an indicator of the importance of using these tools by health authorities in different countries to spread the authentic and reliable information regarding this pandemic. This would assess in reducing the negative psychological impacts of such issues.

Mathematical modeling in the era of COVID-19 has an essential role in understanding the spread of this disease and to set the best policies to minimize its spread in the population [30]. Also, it is important to assess the psychological consequences associated with COVID-19 and the most affected groups to design prevention and education programs [31]. Therefore, the fear of COVID-19 Scale (FCV-19S) was developed to help in the efforts of treating and preventing the spread of this disease [31]–[33]. In the current study, the percentages of depression, anxiety, and stress were lower among Saudi participants when compared to non-Saudi ones. In which, these percentages were 30.40%, 13.51% and 27.70% compared to 50.74%, 34.23% and 59.70%, respectively. However, the high percentages of these issues among non-Saudis could be attributed to the high percentage of females in this group compared to Saudi participants (74.62% vs. 24.32%, respectively). Females are more prone to having these mental health issues related to COVID-19 pandemic as discussed in this part.

The percentages of both anxiety and stress among Saudi participants seem to be lower compared to those reported by Wang et al. (2020) in the general population in China during the initial phase of the COVID-19 epidemic [20]. They found that 36.4% and 32.1% out of 1210 respondents were with different degrees of anxiety and stress using the DASS-21 scale, compared to13.51% and 27.70% in Saudi respondents in our study. However, the percentage of depression among Saudi participants seems to be similar to that reported in that study which detected it in 30.3% of that population compared to 30.4% among Saudi respondents. Liu et al. (2020) examined the behavior changes and psychological status in 608 participants in China during the COVID-19. They detected depression, psychological abnormalities, and phobia in 27.1%, 7.7%, and 10.1% of the respondents [15].

The evaluation of mental health issues during the current pandemic is essential. It can be associated to severe consequences such as suicide [16]–[18],[34]. Also, gender-based suicides should be taken into account when designing mental health care and suicide prevention programs. In this regard, the rates of suicide were reported to be higher in males compared to females in most countries [35],[36]. In the current study, females seem to have significantly higher frequency of depression (56.97%), anxiety (30.23%) and stress (54.65%); compared to 23.25%, 13.17% and 26.35% in males, respectively. These findings are in concordance with the conclusions obtained by Wang et al. (2020). They found that females have higher scores in the DASS depression, anxiety, and stress subscales [20]. Similarly, a study from India during the COVID-19 pandemic concluded that a higher psychological effect is expected with the female gender [37]. Another study from Spain during the COVID-19 pandemic found that women amongst other groups showed worse mental health [38]. Regarding the association between COVID-19 news following and mental health issues; daily following of the pandemic news seems to be associated with an elevated percentages of severe + depression, anxiety, and stress. This seems to be logical, as the continuous following of COVID-19 news mainly the numbers of new cases and deaths could be a continuous trigger. The daily following of the pandemic news was particularly associated with a higher percentage of severe + anxiety taking into account other factors such as gender, age and ethnicity. In this regard, it was mainly among females, non-Saudis and age group (up to 35years). In our study, it was observed that respondents of younger ages (up to 35 years) have higher percentages of depression and stress compared to those of age 36+ years. Huang and Zhao (2020) in their study of certain mental health illnesses during the COVID-19 outbreak in China; reported that younger people were at high risk of having mental illnesses [39]. Also, a study from India during the COVID-19 pandemic concluded that a higher psychological effect is predicted with younger age [37].

In the light of the above findings and information, we recommend that special mental health care programs to be designed to deal with the psychological issues related to COVID-19 pandemic. These programs can use social media and internet as effective tools to reach targeted groups effectively and easily. Special attention should be paid for both females and younger individuals who seem to be particularly affected psychological during this issue. Avoiding excessive following of the pandemic news could help reduce the psychological effects because of the current pandemic. Also, mental health care strategy designed for health care workers might be valuable to minimize the psychological effects in these front lines workers [40].

## 5. Conclusion

Less exposure to the pandemic information and news mainly from unreliable sources such as social media could help in reducing the frequency of mental health issues related to the ongoing pandemic. Special attention and care should be paid to both females and younger people during the current COVID-19 pandemic, as they appear to be especially affected psychologically by it.
