# Supplementary material for: The magnitude of COVID-19 related stress, anxiety and depression associated with intense mass media coverage in Saudi Arabia
Source: AIMS Public Health. 2020 Sep 1;7(3):664–78. doi: 10.3934/publichealth.2020052 (PMC7505795; doi:10.3934/publichealth.2020052)
Supplement: Supplementary file 1 [file publichealth-07-03-052-s001.pdf]

AIMS Public Health

AIMS Public Health, 7(3): 664–678.
DOI: 10.3934/publichealth.2020052.
Received: 30 June 2020
Accepted: 28 August 2020
Published: 01 September 2020

http://www.aimspress.com/journal/aimsph

*Research article*

# The magnitude of COVID-19 related stress, anxiety and depression associated with intense mass media coverage in Saudi Arabia

**Yosef Mohamed-Azzam Zakout[1,3,*], Fayez Saud Alreshidi[2], Ruba Mustafa Elsaid[1] and Hussain Gadelkarim Ahmed[1,3]**

[1] Department of Pathology, College of Medicine, University of Hail, Hail, Kingdom of Saudi Arabia
[2] Department of Family and Community Medicine, College of Medicine, University of Hail, Hail, Kingdom of Saudi Arabia
[3] Department of Histopathology and Cytology, Faculty of Medical Laboratory Sciences, University of Khartoum, Khartoum, Sudan

* **Correspondence:** Email: y.zakout@uoh.edu.sa; yosifzakot@yahoo.com; Tel: +966551369928; +249912927880.

## Supplementary

**The 32 questions of the questionnaire (were translated to Arabic).**

**1-** Nationality:
Saudi
Non-Saudi
**2-** Are you currently living in Saudi Arabia?
Yes
No
**3-** Gender:
Male
Female
**4-** How old are you (in years)?
**5-** Marital status:
Married
Unmarried

**6-** Education level:
Basic study
Graduate
Postgraduate
**7-** Occupation:
Unemployed
House wife
Student
Employee
Self-employed
Retired
Other
**8-** How do you describe your level of following the news of COVID-19 pandemic?
Excessively (Daily)
Actively (4-6 days a week)
Moderately (2-3 days a week)
Rarely (1 day or less weekly)
**9-** What is your primary source of following the news of this pandemic?
T.V
Radio
Social media
Journals
Internet
**10-** Do you feel the extensive coverage in the media of COVID-19 news causes you stress and/or anxiety?
Yes
No
**11-** Do you live alone?
Yes
No
**12-** I found it hard to wind down.
0. Did not apply to me at all
1. Applied to me to some degree, or some of the time
2. Applied to me to a considerable degree or a good part of time
3. Applied to me very much or most of the time
**13-** I was aware of dryness of my mouth.
0. Did not apply to me at all
1. Applied to me to some degree, or some of the time
2. Applied to me to a considerable degree or a good part of time
3. Applied to me very much or most of the time
**14-** I couldn’t seem to experience any positive feeling at all.
0. Did not apply to me at all
1. Applied to me to some degree, or some of the time
2. Applied to me to a considerable degree or a good part of time

3. Applied to me very much or most of the time

**15-** I experienced breathing difficulty (e.g. excessively rapid breathing, breathlessness in the absence of physical exertion).

0. Did not apply to me at all
1. Applied to me to some degree, or some of the time
2. Applied to me to a considerable degree or a good part of time
3. Applied to me very much or most of the time

**16-** I found it difficult to work up the initiative to do things.

0. Did not apply to me at all
1. Applied to me to some degree, or some of the time
2. Applied to me to a considerable degree or a good part of time
3. Applied to me very much or most of the time

**17-** I tended to over-react to situations.

0. Did not apply to me at all
1. Applied to me to some degree, or some of the time
2. Applied to me to a considerable degree or a good part of time
3. Applied to me very much or most of the time

**18-** I experienced trembling (e.g. in the hands).

0. Did not apply to me at all
1. Applied to me to some degree, or some of the time
2. Applied to me to a considerable degree or a good part of time
3. Applied to me very much or most of the time

**19-** I felt that I was using a lot of nervous energy.

0. Did not apply to me at all
1. Applied to me to some degree, or some of the time
2. Applied to me to a considerable degree or a good part of time
3. Applied to me very much or most of the time

**20-** I was worried about situations in which I might panic and make a fool of myself.

0. Did not apply to me at all
1. Applied to me to some degree, or some of the time
2. Applied to me to a considerable degree or a good part of time
3. Applied to me very much or most of the time

**21-** I felt that I had nothing to look forward to.

0. Did not apply to me at all
1. Applied to me to some degree, or some of the time
2. Applied to me to a considerable degree or a good part of time
3. Applied to me very much or most of the time

**22-** I found myself getting agitated.

0. Did not apply to me at all
1. Applied to me to some degree, or some of the time
2. Applied to me to a considerable degree or a good part of time
3. Applied to me very much or most of the time

**23-** I found it difficult to relax.

0. Did not apply to me at all

1. Applied to me to some degree, or some of the time
2. Applied to me to a considerable degree or a good part of time
3. Applied to me very much or most of the time

**24-** I felt down-hearted and blue.
0. Did not apply to me at all
1. Applied to me to some degree, or some of the time
2. Applied to me to a considerable degree or a good part of time
3. Applied to me very much or most of the time

**25-** I was intolerant of anything that kept me from getting on with what I was doing.
0. Did not apply to me at all
1. Applied to me to some degree, or some of the time
2. Applied to me to a considerable degree or a good part of time
3. Applied to me very much or most of the time

**26-** I felt I was close to panic.
0. Did not apply to me at all
1. Applied to me to some degree, or some of the time
2. Applied to me to a considerable degree or a good part of time
3. Applied to me very much or most of the time

**27-** I was unable to become enthusiastic about anything.
0. Did not apply to me at all
1. Applied to me to some degree, or some of the time
2. Applied to me to a considerable degree or a good part of time
3. Applied to me very much or most of the time

**28-** I felt I wasn't worth much as a person.
0. Did not apply to me at all
1. Applied to me to some degree, or some of the time
2. Applied to me to a considerable degree or a good part of time
3. Applied to me very much or most of the time

**29-** I felt that I was rather touchy.
0. Did not apply to me at all
1. Applied to me to some degree, or some of the time
2. Applied to me to a considerable degree or a good part of time
3. Applied to me very much or most of the time

**30-** I was aware of the action of my heart in the absence of physical exertion (e.g. sense of heart rate increase, heart missing a beat).
0. Did not apply to me at all
1. Applied to me to some degree, or some of the time
2. Applied to me to a considerable degree or a good part of time
3. Applied to me very much or most of the time

**31-** I felt scared without any good reason.
0. Did not apply to me at all
1. Applied to me to some degree, or some of the time
2. Applied to me to a considerable degree or a good part of time
3. Applied to me very much or most of the time

**32-** I felt that life was meaningless.

0. Did not apply to me at all
1. Applied to me to some degree, or some of the time
2. Applied to me to a considerable degree or a good part of time
3. Applied to me very much or most of the time
